# Endoplasmic reticulum and mitochondria in diseases of motor and sensory neurons: a broken relationship?

**DOI:** 10.1038/s41419-017-0125-1

**Published:** 2018-02-28

**Authors:** Nathalie Bernard-Marissal, Roman Chrast, Bernard L. Schneider

**Affiliations:** 1 Brain Mind Institute, Ecole Polytechnique Fédérale de Lausanne (EPFL), 1015 Lausanne, Switzerland; 2 Departments of Neuroscience and Clinical Neuroscience, Karolinska Institutet, SE-171 77 Stockholm, Sweden

## Abstract

Recent progress in the understanding of neurodegenerative diseases revealed that multiple molecular mechanisms contribute to pathological changes in neurons. A large fraction of these alterations can be linked to dysfunction in the endoplasmic reticulum (ER) and mitochondria, affecting metabolism and secretion of lipids and proteins, calcium homeostasis, and energy production. Remarkably, these organelles are interacting with each other at specialized domains on the ER called mitochondria-associated membranes (MAMs). These membrane structures rely on the interaction of several complexes of proteins localized either at the mitochondria or at the ER interface and serve as an exchange platform of calcium, metabolites, and lipids, which are critical for the function of both organelles. In addition, recent evidence indicates that MAMs also play a role in the control of mitochondria dynamics and autophagy. MAMs thus start to emerge as a key element connecting many changes observed in neurodegenerative diseases. This review will focus on the role of MAMs in amyotrophic lateral sclerosis (ALS) and hereditary motor and sensory neuropathy, two neurodegenerative diseases particularly affecting neurons with long projecting axons. We will discuss how defects in MAM signaling may impair neuronal calcium homeostasis, mitochondrial dynamics, ER function, and autophagy, leading eventually to axonal degeneration. The possible impact of MAM dysfunction in glial cells, which may affect the capacity to support neurons and/or axons, will also be described. Finally, the possible role of MAMs as an interesting target for development of therapeutic interventions aiming at delaying or preventing neurodegeneration will be highlighted.

## Facts

- Defects in endoplasmic reticulum and mitochondria are observed in multiple forms of neurodegenerative diseases.
- Sites of contacts between endoplasmic reticulum and mitochondria at MAMs play a critical role in normal function of both of these organelles.
- Alteration of MAMs lead to many of the pathophysiological changes observed in neurodegenerative diseases.
- Mutations in genes encoding proteins implicated in MAM function have a causal role in ALS and HMSN.
- Modulation of MAM function can alleviate some symptoms of neurodegeneration.

## Open Questions

- How is the assembly and maintenance of MAMs controlled?
- How do different defects affecting MAMs (e.g. mutation in genes encoding different components of MAMs) lead to alteration in ER/mitochondria function?
- Are the MAMs in the soma and in the axon affected to the same extent by the disease?
- How much are the changes in glial MAMs contributing to pathophysiology of neurodegeneration?

## Introduction

Neuronal function relies on synaptic transmission, which is based on the propagation of action potentials along axons and neurotransmitter release. As the majority of biosynthetic pathways take place in the neuron soma, axons and distal synaptic contacts need efficient axonal transport for the supply of organelles and vesicles. Axonal transport is driven by motor proteins, which consume substantial amounts of energy. Sensory neurons and motoneurons have axons up to 1 m in length. Their extreme dendrite/cell-body/axon polarization and their large soma make these neurons highly demanding in energy to function properly. It was estimated that the anterograde transport of one vesicle along the 1 m long axon of a human motoneuron requires approximately 1.25 × 10^8^ adenosine tri-phosphate (ATP) molecules^1^. High metabolic demand requires a tight coordination between protein secretion, organelle biogenesis, and degradation processes that avoid accumulation of defective components. Long axons are therefore particularly vulnerable to conditions of suboptimal energy supply^2^. The axonal compartment often degenerates first in diseases affecting long-projection neurons, such as amyotrophic lateral sclerosis (ALS) and hereditary motor and sensory neuropathies (HMSNs) also known as Charcot–Marie–Tooth diseases (CMTs)^2,3^.

Maintenance of ionic gradients, as well as the mobilization and cycling of synaptic vesicles in the axons, are mechanisms that are energetically demanding^4^ and require controlled intracellular calcium signaling^5^. This is partly achieved by compartmentalizing biochemical reactions in pools of specialized organelles. The endoplasmic reticulum (ER) is the main site for protein and lipid biosynthesis and intracellular calcium storage, while mitochondria generate most of neuron’s ATP via oxidative phosphorylation. Importantly, the interorganelle communication is essential to coordinate these activities. Mitochondrial ATP production depends on calcium concentration, which is controlled by the ER^6^. Juxtapositions of ER and mitochondrial membranes, called mitochondria-associated membranes (MAMs), represent one of the most specialized sites for interorganelle membrane interactions. ER and mitochondria become dysfunctional early during neuronal degeneration^7,8^. Therefore, defects at the level of MAMs could be among the initial triggers of the disease. For some of the genes linked to neurodegenerative diseases, the encoded proteins are located at MAMs^9^. Furthermore, MAM dysregulation occurs in several neurological pathologies including Alzheimer’s disease, Parkinson’s disease, and motoneuron diseases^10–12^.

In this review, we will summarize the general function of MAMs with a particular attention on their role in neuronal degeneration in ALS and HMSN diseases, and discuss the contribution of MAMs in other cell types that support neuronal function in the central (CNS) and peripheral nervous system (PNS). We will also evoke possible outcomes of this research in terms of therapies targeting MAMs in neurodegenerative diseases.

## Mitochondria–ER contacts

Tight contacts between the ER and the mitochondria were originally observed in 1956 on electron micrographs of the rat liver^13^. The characterization of the MAMs has been refined and now relies, in addition to electron microscopy, on biochemical methods, such as cell fractionation^14,15^, and fluorescent microscopy, in particular using super-resolution light microscopy^16^. Fluorescence *resonance energy transfer*-based indicators of ER–mitochondria proximity^17^ and *in situ* proximity ligation assays^18,19^ have been extensively used to characterize MAMs. Contact sites between ER and mitochondria are defined by an intermembrane distance of 10–30 nm, which depends on cell type and the level of stress to which the cells are exposed^20^. It is estimated that 15–20% of the mitochondrial surface is connected to the ER^21^.

MAMs control various critical cellular functions including lipid exchange, calcium homeostasis, autophagy, and mitochondrial dynamics, and contribute to regulation of inflammation and cell death^10,12^.

**Fig. 1:**
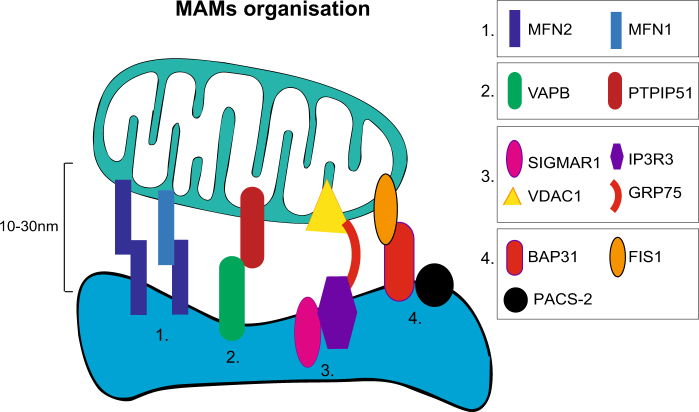
Overview of proteins required for ER–mitochondria tethering The figure summarizes the four main tethers at MAMs. (1) Besides its main location at the outer mitochondrial membrane, a small portion of MFN2 can be found at ER. ER-MFN2/mitochondria-MFN2 homodimers and ER-MFN2/mitochondria-MFN1 heterodimers participate in the tethering of these two organelles. (2) VAPB located at the ER surface interacts with PTPIP51 located at the mitochondrial outer membrane. (3) The IP3R3 at the ER makes close connections with the mitochondrial channel VDAC1 owing to the chaperone GRP75, which interacts directly with the two proteins. At the ER, SIGMAR1 is chaperoning IP3R3. This complex regulates calcium transfer between the two organelles.(4) ER-resident protein BAP31 interact with the mitochondrial fission protein FIS1. With PACS-2, this MAM complex regulates ER–mitochondria coupling and apoptosis.

In mammalian cells, four types of connectors between ER and mitochondria have been identified. We have summarized their implications into MAMs signaling and ALS/HMSN in Tables 1 and 2 and Fig. 1:

**Table 1:** Overview of the main MAMs connector proteins and their involvement in ALS or HMSN diseases

	Identification/role at MAMs	Pathogenic role
Tethering proteins complexes		
MFN1/2	MFN1 and MFN2 are dynamin-related GTPases which contribute to the mitochondrial fusion process^127^. Originally identified on the outer mitochondrial membrane, MFN2 has also been observed on the outside of the ER^22^. Homodimers (MFN2–MFN2) or heterodimers (MFN2–MFN1) can form between the ER and the mitochondria. The p21Ras-binding domain of MFN2 could be important in MFN2–MFN2 interaction and ER–mitochondria coupling^128^. Deletion of MFN2 affects mitochondria–ER contacts. Whether MFN2 is a positive or a negative regulator of MAMs formation is still a matter of debate^17,43–45^	Mutations leading to amino acid substitutions in the MFN2 protein, or causing a decrease in MFN2 level, have been associated to several diseases including CMT2A/HMSN2A^129^, type 2 diabetes, and obesity, as well as Parkinson’s disease^130^
VAPB	VAPB is an integral ER protein, which binds the mitochondrial PTPIP51^23^. Overexpression of VAPB or PTPIP51 increases the number of ER–mitochondria contacts, whereas downregulation of these proteins diminishes those contacts^23,53^	P56S mutation of VAPB causes a familial form of ALS (ALS8), and VAPB–PTPIP51 binding is defective in models of ALS^53^
PTPIP51		
Linkers regulating calcium exchange		
IP3R3	IP3R3 is maintained in close proximity with the mitochondrial VDAC1 by interacting with the GRP75 chaperone which links these two proteins in MAMs^131^. This complex facilitates the transfer of calcium from the ER towards the mitochondrial inter-membrane space^6^. In CHO cells, knockdown of the ER-located protein IP3R specifically reduces mitochondrial calcium content^24^, whereas its stimulation with a selective agonist increases ER calcium flux and calcium uptake by the mitochondria^21^. Knockdown of GRP75 decreases the transfer of calcium between the two organelles^132^	Altered VDAC1 function and/or localization have been observed in ALS and HMSN^33,35,55^
GRP75		
VDAC1		
Linkers implicated in induction of mitochondria-induced apoptosis		
BAP31	Interaction between the ER-resident protein BAP31 and the mitochondrial fission protein FIS1 recruits procaspase-8 and initiates BAP31 cleavage followed by ER calcium release^25^. Resulting increase in mitochondrial calcium can trigger pro-apoptotic release of cytochrome c	The roles of these factors in motoneuron disease and in HMSNs remain to be explored
FIS1		
PACS-2	The knockdown of PACS-2 uncouples mitochondria from the ER. PACS-2 downregulation induces BAP31 cleavage-dependent cell death, which suggests an antiapoptotic role of this factor. Its exact function at MAMs remains unclear^26^	

*ALS* amyotrophic lateral sclerosis, BAP31 B-cell receptor-associated protein 31, *CHO* Chinese hamster ovary, *ER* endoplasmic reticulum, *FIS1* mitochondrial fission 1 protein, *GRP75* 75 kDa glucose-regulated protein, *HMSN* hereditary motor and sensory neuropathy, *IP3R3* inositol 1,4,5 tri-phosphate receptor type 3, *MAM* mitochondria-associated membrane, *MFN1/2* Mitofusin 1 and 2, *PACS-2* phosphofurin acidic cluster sorting protein 2, *PTPIP51* protein tyrosine phosphatase interacting protein 51, *VAPB* vesicle-associated membrane protein-associated protein B, *VDAC1* voltage-dependent anion channel 1

**Table 2:** Overview of the proteins enriched at MAMs and their involvement in ALS or HMSN diseases

Function	Identification/role at MAMs	Pathogenic role
ER chaperones	CRT, CNX, BIP, PDI, and SIGMAR1 are enriched at MAMs^6^. They regulate ER calcium concentration (CRT and CNX) and also promote proper folding of nascent proteins in a calcium-dependent manner^133^. When localized at MAMs, these chaperones represent high-capacity calcium stores that can be readily used when calcium needs to be transferred to the mitochondria^6^. ER chaperones can also stabilize molecular complexes: e.g. SIGMAR1 stabilizes IP3R, preventing its degradation by the proteasome^6^	Function of several ER chaperones is affected in ALS which may contribute to ER stress and motoneuron death^84,133,134^
Autophagy machinery	MAMs could be an important site for autophagosome formation^93,94^. Whether MAMs act as positive or negative regulators of autophagosome formation is still debated. Proteins involved in the early steps of the autophagy process, such as p150, and Vps34, as well as the autophagy-related proteins ATG15 and ATG14L, are located at the ER–mitochondria interface in conditions of starvation^12^. The disruption of ER–mitochondria connections, via MFN2 or PACS-2 knockdown, prevents ATG14 enrollment and subsequent autophagosome formation in starved cells^94^	Autophagy is defective in both ALS and HMSN^100,135^
		VAPB, MFN2, and SIGMAR1 regulate autophagy process^82,93,94^ and are linked to ALS or HMSN^34–36,39,42,136–138^
Mitochondria function, transport, and turnover	Mitochondrial fusion/fission is regulated by a pool of proteins (MFN1, MFN2, Drp1, Fis1, INF2), which participate in ER–mitochondria tethering and/or are localized at the MAMs^31^. The main mitochondrial fission protein, Drp1, forms a helix around the mitochondria to create a constriction ring	MFN2 mutations are linked to CMT2A/HMSN2A^39,42,138^
	ER may participate in mitochondrial fission by physically encircling the mitochondria at MAMs^139^. This step relies on actin polymerization, a process which appears to be initiated by the ER factor INF2^140^	INF2 heterozygous mutations are linked to CMT2D complicated by glomerulopathy^141^
	Key regulators of mitochondrial axonal transport are also associated with the MAMs. The MIRO proteins regulate mitochondria mobility by connecting the organelles with the motor proteins kinesin and dynein. A helix–loop–helix structural domain called EF-hand motif present in the MIRO isoforms 1 and 2 functions as calcium sensor. A change in calcium level induces conformational changes in the complex, leading to the detachment of MIRO from the microtubules^142^	Heterozygous missense mutations of the *DNM1L* gene encoding DRP1 leads to severe neonatal encephalopathy combined or not with epilepsy^143^
Lipid synthesis	Enzymes involved in the synthesis of phospholipids (PS synthase 1 and 2), cholesterol, and sphingolipids (ceramide) are enriched at MAMs. Some lipids, such as PS, are transferred from the ER to the mitochondria to be transformed to PE^30^. Mitochondrial membranes are enriched in PE, which is crucial for mitochondrial function and cell survival. The distribution of lipids in membranes forms specialized areas such as lipid rafts, and thereby modulates the recruitment of proteins at MAMs such as SIGMAR1^144^.	The role of these factors in disease affecting motor or sensory neurons remains to be explored
Oncogenes and tumor suppressors	The tumor suppressors PML, p53, and protein phosphatase and tensin homolog deleted on chromosome 10 (PTEN) localize to MAMs. They control ER/mitochondria calcium signalling by modulating the level of IP3R phosphorylation^27,28^. At MAMs, the oncogenes HRas and KRas control calcium transfer from ER towards mitochondria and maintain calcium homeostasis during cancer progression^145^	P53 levels are increased in ALS spinal cord motoneurons^146^. PTEN downregulation partially protects ALS motoneurons *in vitro*^147^
Inflammasome	In the inflammasome, NLRP3 serves as a platform for caspase 1 activation and maturation of pro-inflammatory cytokines such as IL1β. NLRP3 re-localizes from the ER to MAMs upon inflammation, a process that could be dependent on mitochondrial ROS production^29^	Inflammasome components are mainly expressed in astrocytes and microglia, in the spinal cord of SOD1 ALS mice and ALS patients^148,149^

*ALS* amyotrophic lateral sclerosis, *BIP* binding immunoglobulin protein (also known as HSPA5, heat-shock protein family A (Hsp70) member 5), *CMT* Charcot–Marie–Tooth disease, *CRT* calreticulin, *CNX* calnexin, *ER* endoplasmic reticulum, *HMSN* hereditary motor and sensory neuropathy, *IL* interleukin, *INF2* inverted formin-2, *MAM* mitochondria-associated membrane, *MFN1/2* mitofusin 1/2, *MIRO* mitochondrial Rho GTPase, *NLRP3* nucleotide-binding oligomerization domain, leucine-rich repeat and pyrin domain containing 3, *PDI* protein disulfide isomerase, *PE* phosphatidylethanolamine, *PML* promyelocytic leukemia, *PS* phosphatidylserine, *PTEN* phosphatase and tensin homolog deleted on chromosome 10, *ROS* reactive oxygen species, *SIGMAR1* sigma non-opioid intracellular receptor 1, *SOD1* superoxide dismutase 1

(1) Initially observed at the mitochondria, mitofusin 2 (MNF2) is a dynamin-related GTPase identified at the ER surface almost 10 years ago^22^. MFN2 contributes to ER and mitochondria tethering either by homologous interaction between ER-associated MFN2 and mitochondrial MFN2 or by heterologous interaction with mitofusin 1 (MFN1), a homolog protein only located at the outer mitochondrial membrane^22^.

(2) The second tethering complex relies on vesicle-associated membrane protein-associated protein B (VAPB), an ER protein binding the mitochondrial protein tyrosine phosphatase-interacting protein 51 (PTPIP51)^23^.

(3) The subtype 3 of the 1,4,5-triphosphate receptor (IP3R3) forms a complex with the 75 kDa glucose-regulated protein (GRP75) and the mitochondrial voltage-dependent anion channel 1 (VDAC1), which serves as a calcium exchange platform between ER and mitochondria. These three proteins are referred to as tethering and calcium signaling proteins at MAMs^24^.

(4) The ER-resident protein BAP31 (B-cell receptor-associated protein 31), the mitochondrial fission protein FIS1 (fission 1 homolog) and the phosphofurin acidic cluster sorting protein-2 (PACS-2) are MAM connectors involved in the induction of apoptosis^25,26^.

In addition, ER chaperones, autophagy-regulating factors, proteins involved in mitochondrial dynamics, enzymes involved in lipid synthesis, oncogenes, and tumor suppressors as well as the inflammasome NLRP3 (nucleotide-binding oligomerization domain, leucine-rich repeat and pyrin domain containing 3) are enriched at MAMs (Table 2), where they participate in MAMs signaling^6,12,27–30^.

## MAM dysfunction in ALS and HMSN diseases

**Fig. 2:**
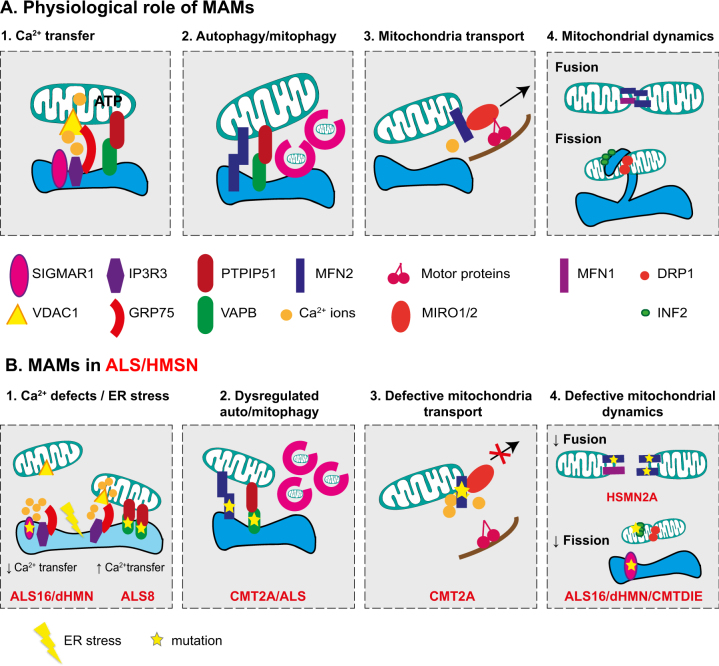
Overview of the physiological roles of MAMs and potential consequences of ALS and HMSN mutations on MAM function **a** Summary of MAM proteins and their role in the maintenance of long-projecting neuron physiology. Boxes 1, 2, 3, and 4 describe the key functions handled at MAMs. (1) Calcium transfer between ER and mitochondria is mainly controlled by the IP3R3–GRP75–VDAC1 complex. Through its interaction with IP3R3, SIGMAR1 prevents IP3R3 degradation, thus maintaining calcium flux. (2) MAMs have been recently considered as a site of autophagosome formation. VAPB–PTPIP51 as well as MFN2 have been shown to regulate this process at MAMs. (3) Mitochondrial transport can be controlled at MAMs by the means of interactions between MFN2 and the MIRO1/2 proteins. MIRO proteins connect to the motor proteins kinesin and dynein in a calcium-dependent manner. (4) MAMs appear as a site-promoting fusion/fission. These processes are mainly controlled by MFN2, MFN1, DRP1, or INF2. Several proteins involved in mitochondrial dynamics such as MFN2, DRP1, or INF2 are located at MAMs. The association of DRP1, INF2, and ER tubules create a constriction ring around mitochondria favoring its fission. **b** Potential consequences of ALS and HMSN mutations in MAM proteins on function of long-projections neurons. Mutations in genes encoding the MAM proteins SIGMAR1, VAPB, and MFN2 have been identified in ALS or HMSN patients. This figure illustrates defects that could occur in long-projection neurons as a consequence of alteration in MAM functions. (1) Mutations in SIGMAR1 lead to loss of interactions between IP3R3 and VDAC1. Consequently, calcium transfer towards mitochondria is impaired leading to an increase of cytosolic calcium. On the contrary, mutations in VAPB reinforce its connection with PTPIP51, increasing mitochondrial calcium content as well as cytosolic calcium. In the context of both mutated SIGMAR1 and VAPB, deregulation in calcium levels impairs ER function causing ER stress. (2) Impairment of ER–mitochondria connections related to mutated MFN2 or VAPB could either promote or decrease autophagosome formation. In motoneurons differentiated from iPS cells derived from CMT2A-R94Q patients autophagic flux is increased. Whether mutated VAPB could enhance or decrease autophagy is unknown. (3) Deregulation of calcium levels due to MAM disconnection detaches MIRO proteins from the kinesin/dynein motor proteins. Consequently, mitochondrial transport is impaired. (4) Mutations in MFN2 make MFN2–MFN2 interactions non-functional leading to a reduction in fusion process. In ALS16 or distal HMN patients, loss of contacts between ER and mitochondria in neurons could prevent ER to physically enroll the mitochondria to promote fission. Mutations in INF2 can impair the step of actin polymerization required for mitochondria constriction.

Owing to their central position between ER and mitochondria, there is an increasing interest in the possible role of MAMs in neurodegenerative diseases. In the CNS, changes in MAMs were reported in multiple types of common neurodegenerative diseases, including Alzheimer’s disease, Parkinson’s disease, and spastic paraplegia^31^. In the PNS, motor and sensory neurons have particularly long axons. They represent extreme examples of how important the maintenance of ER and mitochondrial functions can be to sustain high axonal metabolic demand^3^. We will focus on ALS and HMSN, diseases affecting neurons projecting to the periphery, to discuss the implications of dysregulated MAMs in neuronal/axonal pathology^16,32^ (Fig. 2).

### Disturbance of ER–mitochondria connections in ALS and HMSN

Missense mutations in *VAPB* and *SIGMAR1* (sigma non-opioid intracellular receptor 1) can lead to inheritable forms of ALS and HMSN, whereas missense mutations in *MFN2* cause a form of HMSN described as CMT2A (see Box 1). These genes provide the most direct evidence for the role of MAMs in neuron function.

#### Box 1 ALS and HMSN diseases: clinical features and genetics

- *Amyotrophic lateral sclerosis*:
  ALS is an incurable neurodegenerative disease, with a life expectancy of an average of 3–5 years after diagnostic. ALS affects upper motoneurons located in the motor cortex and lower motoneurons in the brainstem and spinal cord. Depending on the first symptoms observed, ALS is classified as of bulbar or spinal onset. ALS patients develop progressing muscle atrophy and paralysis leading to death caused by respiratory failure. ALS is a rare disease with a prevalence around 4–6/100,000. Most of the ALS cases are considered as sporadic (sALS), whereas ~10% are familial (fALS) cases, caused by inherited mutations in one of the fALS genes. Around 15% of ALS cases also present frontotemporal lobar dementia (FTLD), which is characterized by abnormalities in behavior and/or language^150^.
- *Hereditary motor and sensory neuropathy*:
  HMSN, also called Charcot–Marie–Tooth (CMT) disease, represents an heterogeneous group of inherited disorders where motor and/or sensory functions are affected. HMN refers to a pure MN defects and HSN to a pure sensory dysfunction. Usually, distal parts of the body are the first to be affected, with potential progressive spread towards proximal territories. HMSN have a prevalence of 1/2500; the survival of patients is not affected. HMSN is classified according to the mode of inheritance (autosomal, recessive, X-linked), electrophysiological features and predominance of motor and/or sensory symptoms. The two main forms of HMSN are either *demyelinating* (HMSN1 or CMT1, with reduced nerve conduction velocities, <38 m/s in the upper extremities) involving mutations in genes controlling Schwann cell function; or *axonal* (HMSN2 or CMT2, normal nerve conduction velocities, and a decrease in compound muscle action potential amplitudes) with mutations in genes affecting axonal function. For an extensive description of HMSN refer to previous reviews^50,151^. Here, we focus on CMT2 which represents around 20–30% of all CMTs and which implicates MAMs dysfunction.
- Description of the genes discussed in the review and their implications in ALS/HMSN diseases.
*If not specified otherwise, mutations in the genes described below have autosomal-dominant inheritance.*
  SOD1 (superoxide-dismutase 1), which encodes a ubiquitous enzyme catalyzing the transformation of superoxide into hydrogen peroxide and dioxygen, is the first gene identified as mutated in ALS. More than 150 mutations identified in the SOD1 gene are responsible for 20% of fALS (Rosen, 1993)152. Several ALS mouse models are based on the overexpression of mutated human SOD1. Mice overexpressing G93A SOD1 is the best-characterized model of the disease.
  *VAPB* (vesicle-associated membrane protein-associated protein B) mutations are linked to development of ALS8 as well as familial adult spinal muscular atrophy^136^. The VAPB protein is located at the ER and Golgi membrane.
  *TARDBP* (TAR DNA-binding protein gene) mutations (>40 were identified) have been linked to fALS and sALS with or without frontotemporal lobar degeneration (FTLD), and classified as ALS10. TARDBP encodes the protein TDP43 (TAR DNA-binding protein 43), a ubiquitous protein mainly localized in the nucleus where it regulates RNA processing. Accumulations of wild-type TDP43 have been observed in many cases of sALS and FTLD and are considered as a histopathological hallmark of the disease^153,154^.
  FUS (fused in sarcoma) mutations (>30 were identified) are linked to classical ALS (referred to as ALS6) and to a small number of ALS-FTLD. FUS is a RNA-binding protein. Once mutated, both TDP-43 and FUS proteins form toxic cytoplasmic aggregates and are likely to induce the gain of a toxic function^155–157^.
  *C9ORF72* (the chromosome 9 open reading frame 72): the mutated form of this gene contains an intronic hexanucleotide repeat expansion which leads to abnormal expression of C9ORF72-derived transcript in 40% of fALS and 7% of sALS. This transcript also leads to the aberrant expression of dipeptides by repeat associated non-AUG translation. C9ORF72 mutations are associated with pure FTLD or ALS-FTLD. The function of the cytoplasmic protein C9ORF72 is unknown but pre-mRNA containing the expansion forms nuclear foci in neurons of the frontal cortex and spinal cord of ALS patients^158,159^.
  SIGMAR1 (sigma-1 receptor) recessive mutations were identified in both adult-onset^137^ and juvenile-onset ALS (ALS16)^33,36^ and also in patients affected with distal HMN (dHMN)^34,35^. SIGMAR1 is an ER chaperone protein present in MAMs and strongly expressed in motoneurons^38^^,160^. The mutated SIGMAR1 protein is unstable and non-functional, probably acting in a loss-of-function manner^36^.
  MFN2 (mitofusin-2) mutations (>60 were identified) are linked to the development of classical HMSN (also known as CMT2A)^39,42,138^. Mutated MFN2 accounts for ~30% of axonal CMTs. CMT2A patients develop the disease with an early or late onset. The sooner symptoms will appear the more severe they will be. Most of CMT2A patients are severely affected and they become non-ambulatory before the age of 20^50^. More rarely, MFN2 mutations can also lead to optic atrophy and CNS impairment.
  GDAP1 (ganglioside-induced differentiation-associated protein 1) mutations are linked to both axonal and demyelinating CMT known as CMT2K (autosomal dominant, axonal CMT)^49^, CMT4A (autosomal recessive, demyelinating CMT), and AR-CMT2K (autosomal recessive, axonal CMT)^47^^,48^. The onset and symptoms are variable and can involve classical CMT symptoms with variable severity, as well as vocal cord paresis or pyramidal involvement^50^. GDAP1 protein regulates mitochondrial network by promoting mitochondrial fission.
  INF2 (inverted-formin 2) mutations cause intermediate Charcot–Marie–Tooth disease E (CMTDIE), which can be associated with glomerulopathy^141^. One isoform of INF2 localizes at the ER and at MAMs. INF2 protein regulates actin polymerization^31,140^.

De Vos et al.^23^ were the first to demonstrate MAM dysfunction in ALS. They observed that P56S-mutated VAPB, which is linked to ALS8, has increased binding capacity to PTPIP51. Other studies identified loss-of-function homozygous mutations of *SIGMAR1* in familial cases of juvenile ALS (ALS16) and HMSN^33–36^. Downregulation or loss of SIGMAR1 in neurons decreases ER–mitochondria association^18,36^. The loss of contacts between ER and mitochondria is due to the inability of mutated SIGMAR1 to properly bind IP3R3, causing IP3R3 destabilization^18,36^. SIGMAR1 is expressed throughout the CNS, with highest levels in motoneurons of the brainstem and spinal cord^37^. In alpha motoneurons, SIGMAR1 is mainly expressed in subsurface cisternae formed by the ER in postsynaptic densities associated with cholinergic synapses (C terminals). In ALS tissues, SIGMAR1 is abnormally distributed and accumulates in large C terminals, cytoplasm, and proximal axons^38^. Consistent with the possible pathogenic effects of SIGMAR1 loss-of-function, the knockdown of Sigmar1 in mice leads to a reduction in ER–mitochondria contacts in motoneurons, concomitant with impaired locomotion and motoneuron degeneration^18^.

The most common axonal form of HMSN, referred to as CMT2A, is linked to mutations in the *MFN2* gene^39–42^. Although the loss of MFN2 perturbs the number of ER–mitochondria contacts^17,22,43–45^, it is unclear what are the effects of CMT2A-causing dominant mutations on the formation of MAMs. Expression of mutated R94Q MFN2 in MFN2-null cells rescues mitochondrial morphology by complementing the effects of MFN2 on mitochondrial dynamics^22^. However, no changes in the number of MAMs were observed in these cells despite the loss of MFN2^22^. This observation suggests that this CMT2A-causing mutation is unlikely to affect mitochondrial dynamics, whereas pathogenic effects on MAM function remain entirely possible. Overexpression of the homologous protein MFN1 in primary sensory neurons partially decreased axonal degeneration induced by R94Q MFN2^46^. As MFN1 is localized on the mitochondria and not on the ER, it is likely that MFN1 cannot fully restore MAM function, which may explain why the rescue is only partial. Further studies in models of CMT2A will help to determine how MFN2 mutations affect MAM function.

In 2013, Poston et al.^9^ performed a proteomic analysis of MAMs isolated from the mouse brain and identified more than one thousand associated proteins. Among them, ganglioside-induced differentiation-associated protein 1 (GDAP1), originally known to be located on the outer membrane of mitochondria, was found enriched in MAMs. Mutations in *GDAP1* are linked to CMT, which are either recessive and demyelinating (CMT4A), or dominant/recessive and axonal (CMT2K)^47–50^. Gdap1 knockout mice develop peripheral neuropathy^51^. Although there is evidence that the lack of GDAP1 can perturb calcium homeostasis and affect both ER and mitochondria, the role of GDAP1 at the level of MAMs remains unclear^52^.

Other proteins more commonly implicated in ALS, such as superoxide dismutase 1 (SOD1), TAR DNA-binding protein 3 (TDP43), and fused in sarcoma (FUS) (Box 1), do not have any established physiological role in the MAMs. However, pathogenic mutations can confer novel properties to these proteins that can affect MAM function. Compared to their wild-type counterparts, mutated forms of SOD1, TDP43, or FUS proteins tend to accumulate in ectopic compartments in disease conditions, and can perturb MAM function^36,53,54^.

By performing subcellular fractionation in samples from a neuronal cell line overexpressing mutated SOD1 and from the spinal cord of SOD1 ALS mice, Watanabe et al.^36^ found different forms of mutated SOD1 enriched in both the mitochondrial and MAMs fractions, which was not observed for wild-type SOD1^36^. Moreover, the ER-associated mitochondrial surface was decreased in ALS^36^. Previous studies have shown that misfolded mutated SOD1 could bind VDAC1 in spinal cord tissue of ALS rats, reducing the activity of this mitochondrial anion channel^55^. It remains to be explored whether this interaction also prevents complex formation with the grp75 chaperone and IP3R3 at the level of the ER.

Wild-type or mutated forms of TDP43 and FUS overexpressed in cell lines reduce ER–mitochondria contacts by disrupting VAPB-PTPIP51 binding^53,54^. In mice, overexpression of wild-type FUS leads to an ALS phenotype, with progressive hindlimb paralysis and death at 3 months of age^56^. In this model, the number of ER–mitochondria contacts is reduced and VAPB–PTPIP51 binding decreased. However, there is no reported direct binding of TDP43 and FUS to VAPB or PTPIP51 which may explain these effects^53,54^.

Previous work suggested that both TDP43 and FUS activate the glycogen synthase kinase-3b (GSK3B)^57^. GSK3B activity is mainly regulated by inhibitory phosphorylation at serine 9. As phosphorylation controls protein–protein interaction, it was proposed that FUS and TDP43 may regulate VAPB–PTPIP51 dissociation by modulating GSK3B activity, via changes in the level of serine 9 phosphorylation^53,54^. However, the role of GSK3B in MAMs is still unknown. Moreover, these observations did not refer to any specific toxic effect of ALS-causing mutations, as the wild-type forms of TDP43 and FUS also affect MAM formation. Nevertheless, TDP43 and FUS are predominantly localized in the nucleus in physiological conditions, whereas only a small fraction of these proteins reside in the cytoplasm^58–61^. Pathogenic conditions including ALS-causing mutations increase the cytoplasmic expression of TDP43 and FUS^62^, which may cause toxicity at MAMs. Indeed, forced expression of wild-type FUS in the cytoplasm worsened its effect on the MAMs^54^.

Overall these studies highlight MAMs as a common starting point in motoneuronal degeneration caused by several genes associated to ALS and HMSN. A recent interactome study performed in a neuronal cell line expressing wild-type chromosome 9 open reading frame 72 (C9orf72) shows its enrichment in the mitochondria-enriched fraction^63^. It was found to interact with several proteins located on the mitochondrial outer membrane, including known components of the MAMs. Future research should address whether hexanucleotide repeat extensions in the *C9ORF72* gene, which cause about 30% of familial ALS, may also affect MAM function.

## Pathophysiological consequences of disrupted MAMs

MAMs participate in the regulation of crucial cellular functions such as calcium homeostasis, lipid production, autophagy, mitochondrial dynamic, and motility, as well as axonal maintenance. These functions are often defective in ALS/HMSN, hence contributing to motoneuron dysfunction and degeneration. Here, we review these findings, further underlining the possible contribution of MAMs to these processes (Fig. 2).

### Axonal degeneration and MAMs

Since most of the studies focused on the role of MAMs in the cell body, there is scarce information regarding their role in the axonal compartment. With respect to motoneuron disease, it is unknown if MAM dysfunction may specifically affect the axons since most studies have used cell lines with no or limited axonal compartment. Furthermore, it is technically challenging to visualize MAMs in axons, where the interaction between the ER and the mitochondria are likely to be highly dynamic, to allow for effective axonal transport of mitochondria. Nevertheless, both the rough and smooth ER as well as mitochondria can be visualized by electron microscopy and immunofluorescence in the axoplasm of sciatic nerve^64,65^. Using electron microscopy combined with three-dimensional reconstruction, Villegas et al.^66^ noticed the presence of mitochondria closely associated to the smooth ER in sciatic nerve explants. Using the sciatic nerve explant as an *in vitro* model of injury-induced axonal degeneration, they found that modulating calcium transfer from the ER towards the mitochondria protects against axonal degeneration^66^.

In context of ALS and HMSN, mutations in the MAM-associated proteins SIGMAR1 and MFN2 induced axonal degeneration, suggesting that MAMs are indeed important for axon maintenance^18,46^. Moreover, inhibition and deletion of SIGMAR1 similarly decreased MAMs in the axon and soma of neurons^18^. Importantly, axonal degeneration precedes cell body degeneration in models of ALS and HMSN that are related to MAMs defects^18,46,67^. Therefore, one can hypothesize that in long-projection motoneurons, the axonal compartment is more vulnerable to MAM dysfunction than the neuronal cell body. However, further research is warranted to address this question.

### Calcium dyshomeostasis

Dysregulation of calcium homeostasis is thought to play a key role in neuronal degeneration in both ALS and HMSN diseases. Local calcium concentrations are tightly regulated inside the cell to avoid calcium overload in the ER and mitochondria, which can trigger apoptosis^68^. By bringing ER and mitochondria in close proximity, MAM function as a platform for the exchange of calcium between these two organelles. MAMs have an important role in controlling calcium levels in the ER and also in mitochondria necessary to produce ATP.

Disruption of key factors, including VAPB–PTPIP51, SIGMAR1, and MFN2, correlates with perturbations of calcium homeostasis^18,23,46,69^. Downregulating either VAPB or PTPIP51 by RNA interference in cell lines reduces the number of ER–mitochondria contacts, which affects the mitochondrial uptake of calcium released from ER stores^23^. Unlike wild-type VAPB, expression of P56S-mutated VAPB reinforces ER–mitochondria interactions, which causes an increase in calcium transferred to the mitochondria^23^. In primary cortical neurons, overexpression of P56S VAPB also perturbs resting cytosolic calcium levels, which affects the anterograde transport of the mitochondria^69^. It is however unclear if changes at the level of ER–mitochondria contacts are induced in the latter case.

Similarly, changes in SIGMAR1 activity affect the cytosolic and mitochondrial levels of calcium *in vitro*^18,36,38^. The physiological activity of SIGMAR1 regulates intracellular calcium levels in motoneurons. In embryonic motoneurons, inhibition of SIGMAR1 with the selective antagonist NE-100 increases intracellular resting calcium levels and prolongs the time needed for basal calcium levels to recover after potassium-evoked depolarization^18^. On the other hand, overexpression of SIGMAR1 facilitates mitochondrial calcium uptake^36^. However, similar to SIGMAR1 downregulation by siRNA, the expression of two ALS-linked SIGMAR1 mutants (E102Q, L95fs) in neuronal cell lines causes an increase in cytoplasmic calcium levels and reduces mitochondrial calcium levels following ATP stimulus^36,38^. These results indicate that the loss of SIGMAR1 activity due to ALS-associated mutations may affect motoneuron function and survival via perturbations of calcium homeostasis at the level of MAMs.

Abnormal calcium handling was also linked to HMSN. Despite the implication of MFN2 in HMSN, most of the results on the role of MFN2 in MAM function were obtained by modulating its expression in cell lines. Only few experiments have been performed in neurons^22^. Recent studies have used lentivirus-transduced cultures of sensory neurons to compare the effects of wild-type MFN2 and the CMT2A-associated R94Q mutant following overexpression. Overexpression of mutated MFN2 caused axonal degeneration, which was concomitant with an axonal calcium rise^46^. However, it remains to be determined whether elevated calcium levels induced by mutated MFN2 are due to MAM dysfunction.

Conversely, the level of intracellular calcium can also affect ER–mitochondria interactions. Massively releasing calcium from the ER compartment using thapsigargin leads to ER mitochondria detachment, which may avoid further rise of calcium inside the mitochondria and prevent pro-apoptotic effects^70^.

#### MAM dysfunction affects the transport and dynamics of mitochondria

In neurons, communication between the cell body and synaptic terminals is based on axonal transport. In particular, efficient axonal transport is key for the mitochondria to reach specialized sites with high-energy demand, such as the nodes of Ranvier or the synapses^71^. In models of motoneuron diseases, defects of mitochondrial axonal transport have been observed both *in vitro* and *in vivo*^18,46,72–74^. Interactions between ER and mitochondria are likely to occur in these compartments, and defects in several MAM-associated proteins have been shown to impair axonal transport.

Morotz et al.^69^ demonstrated that overexpression of the ALS-associated P56S VAPB mutant affects the anterograde axonal transport of mitochondria in neurons. Concomitant to these defects, the level of tubulin-associated MIRO1 is reduced. MIRO1/2 are calcium-sensitive Rho-like GTPases located at MAMs, linking mitochondria to kinesin^74^ (see Table 2). P56S VAPB leads to an increase in cytoplasmic calcium levels, which could release the MIRO1/trafficking kinesin protein 1/kinesin-1 complex from the microtubules and decouple mitochondria from axonal transport^75^. Importantly, when MIRO1 is overexpressed in neurons expressing P56S VAPB, the association of MIRO1 with tubulin is rescued and mitochondrial transport restored^69^.

Mitochondrial axonal transport defects were also reported in primary sensory neurons following either Mfn2 deletion or overexpression of the CMT2A-associated R94Q mutant^46,74,76^. Both anterograde and retrograde transports were slower, with mitochondria spending more time paused^74^. These defects are similar to the ones observed following knockdown of MIRO2, suggesting that MIRO2 function may be altered in CMT2A neurons. Despite the fact that the R94Q MFN2 may still interact with the MIRO-associated transport machinery, calcium handling could be altered in these axons, causing pathological changes in the association of MIRO proteins to the microtubule tracts^46,74^. In transgenic mice overexpressing R94Q MFN2, mitochondria accumulate in the distal part of the nerve, reinforcing the notion that the axonal transport of mitochondria could be affected^77^. However, other studies suggest that general axonal transport defects are not the sole cause of HMSN. When decreasing general axonal transport by overexpressing syntaphilin, a protein docking mitochondria to microtubules, sensory neurons do not undergo axonal degeneration^46^. Other CMT2A-associated MFN2 mutations also failed to induce similar axonal transport defects in sensory and motoneurons. In particular, sensory neurons isolated from homozygous or heterozygous R94W Mfn2 knock-in mice show mitochondria velocities comparable to their wild-type counterparts^78^. Two recent studies characterized motoneurons differentiated from iPS cells derived from CMT2A-R94Q patients^79,80^. Surprisingly, mitochondrial trafficking was only mildly affected in these motoneurons and neither axonal elongation nor motoneuron survival were reduced^79,80^, suggesting that mutated MFN2 may cause other pathogenic effects than changes in axonal transport. Moreover, MFN2 mutations could differentially impact on sensory and motor neurons^3^.

The main function of mitochondrial MFN2 is to control the fusion of outer mitochondrial membranes. In neurons overexpressing mutated MFN2, there is an overrepresentation of smaller mitochondria, which may indicate defective fusion^46,81^. The balance between organelle fission and fusion is important for mitochondrial health, and alterations of these dynamic processes can also impact on mitochondrial transport.

Finally, mitochondrial axonal transport is also affected in motoneurons deleted for SIGMAR1 or treated with a selective SIGMAR1 antagonist. Loss of SIGMAR1 specifically affects the retrograde transport of mitochondria, leading to an accumulation of mitochondria in the distal part of axons^18^. It also leads to an increase of mitochondrial length, which contrasts with the effects of R94Q MFN2, but may indicate defects in mitochondrial dynamics. As mentioned above, MAMs have been identified as sites promoting mitochondrial fission. By disrupting ER–mitochondria contacts, the loss of SIGMAR1 could therefore prevent mitochondrial fission.

#### MAMs regulate ER function in ALS/HMSN

In addition to their effects on MAMs, loss of MFN2 or SIGMAR1 and overexpression of P56S VAPB affect ER morphology^22,23,82^. As previously discussed, alteration of MAMs can affect ER calcium homeostasis depending on the rate of calcium transfer between the two organelles. Consequently, the overloading/depletion of calcium inside the ER can negatively impact on protein folding, leading to ER stress associated to the activation of the unfolded protein response (UPR). UPR-mediated intracellular signaling coordinates the suppression of protein synthesis with a transcriptional response enhancing protein folding and ER-associated protein degradation (ERAD) to re-establish ER homeostasis^83^. Alternatively, UPR can also lead to apoptosis if ER function is not restored. Several studies demonstrated the activation of ER stress and UPR in ALS, which was proposed to play a key role in motoneuron degeneration^8,18,84–86^. The depletion of the MAM proteins MFN2, SIGMAR1, and PACS-2, as well as the expression of mutated VAPB, induced UPR in various cell types^18,26,38,87–89^. MFN2, SIGMAR1, and VAPB modulate, by direct interaction, UPR sensors including activating transcription factor 6, inositol-requiring enzyme-1, and protein kinase RNA-like endoplasmic reticulum kinase (PERK)^88,90,91^.

Several components of the UPR machinery and ER chaperones are enriched at MAMs^89,92^. Previous studies have shown that ER and mitochondria can reciprocally regulate their morphology and function via the MAMs. MFN2 acts upstream of PERK by inhibiting this UPR sensor through physical interaction^88^. Conversely, the loss of MFN2 promotes constitutive activation of PERK and associated cell death. Silencing of PERK restores mitochondrial calcium levels as well as mitochondrial elongation. Also, by alleviating ER stress with Salubrinal, it is possible to rescue mitochondrial dynamics following deletion or inhibition of SIGMAR1 and thereby prevent motoneuron degeneration^18^.

#### MAMs and the control of autophagy and mitophagy in models of ALS/HMSN

Autophagy plays an important role in the maintenance of cellular homeostasis and function, by allowing the degradation of long-lived proteins and organelles through lysosomal activity. Autophagy is initiated by the formation of a phagophore, which elongates into a double-membrane structure to form the autophagosome. ER–mitochondria contacts are critical sites for the emergence of autophagosomes, a process controlled by VAPB–PTPIP51, PACS-2, and MFN2^93,94^. However, these factors appear to have different effects in cellular models of autophagy induction. The loss of either VAPB or PTPIP51 leads to the loss of MAMs while stimulating autophagic flux, whereas the downregulation of MFN2 or PACS-2, which also leads to the loss of MAMs, prevents autophagosome formation^93,94^. This apparent discrepancy could be due to different cellular responses depending upon autophagy inducers. However, it may as well indicate that differences exist in the role of these proteins as regulators of autophagy at MAMs.

Dysregulation of ER–mitochondria contacts caused by the dysfunction of MAM proteins including MFN2, SIGMAR1, VAPB, and PTPIP51 may affect autophagic activity, thereby contributing to disease. The presence of inclusions and the accumulation of protein aggregates and defective mitochondria in ALS suggest impairment of degradation systems^95^. Multiple ALS-associated genes, including sequestosome 1 (also known as the ubiquitin-binding protein p62) (*SQSTM1*), optineurin (*OPTN*), ubiquilin 2 (*UBQLN2*), and valosin containing protein (VCP), encode proteins regulating autophagy processes^96–99^.

Similarly, several HMSN-related genes are also known to perturb the autophagy pathway^100^. Loss of SIGMAR1 in the motoneuronal cell line NSC-34 leads to accumulation of autophagic vacuoles, together with markers of autophagy initiation and endolysosomal pathway^82^. Experiments on human fibroblasts demonstrated that lipid rafts at MAMs serve as a platform to induce autophagosome formation^101^. Interestingly, SIGMAR1 depletion can destabilize lipid rafts^82^, which could explain how this factor affects autophagy.

Rizzo et al.^79^ observed a global reduction of the mitochondria content in induced pluripotent stem cell-derived CMT2A motoneurons, with increased expression of mitophagy genes, such as PTEN-induced putative kinase 1 (*PINK1*), Parkin RBR E3 ubiquitin protein ligase (*PARK2*), BCL2 interacting protein 3 (*BNIP3*), and a splice variant of beclin 1 (*BECN1*). These results indicate a potential imbalance in mitochondrial homeostasis, with an enhancement of mitophagy, which is a selective form of autophagy^79^. PINK1/Parkin-dependent ubiquitination of proteins located on the outer mitochondrial membrane targets dysfunctional mitochondria for degradation. Parkin was reported to be located at MAMs, where it promotes calcium transfer by increasing contacts between the two organelles^102^. Parkin interacts with GRP75 and MFN2, and ubiquitinates MFN^31,103,104^. Further studies will be required to determine whether this is affected by MFN2 mutations causing CMT2A.

Additional factors present at MAMs regulate mitophagic activity. PINK1 and BECN1 have been found localized at MAMs after induction of mitophagy in a neuroblastoma cell line^105^. They both promote ER–mitochondria interactions supporting the formation of omegasomes (membrane extensions within the ER), and subsequently autophagosomes^105^. Moreover, PINK1 level is increased in a calcium-dependent manner following mitophagy induction^106^. As calcium is transferred between organelles at MAMs, it is possible that local rises in calcium levels induce PINK1 expression and promote mitophagy. Both Parkin and PINK1 were found to be deregulated in tissues from ALS mice and/or patients^107–109^. Together, there is accumulating evidence for the role of MAMs in controlling mitophagy, which could be related to both ALS and HMSN.

#### Role of MAMs in non-neuronal cells potentially implicated in ALS/HMSN

Multiple studies have highlighted the role of non-neuronal cells in both ALS and HMSN pathologies^110,111^. Besides physical support to neurons, glial cells such as astrocytes, the myelinating oligodendrocytes (OL) and Schwann cells (SCs), also provide metabolic and trophic support^112–116^. It is therefore plausible that the dysregulation of MAM function in glial cells also contributes to pathophysiology of ALS and HMSN. OL and SC produce myelin, which is enriched in lipids that are produced by the ER, such as galactosylceramide^117,118^. The MAM protein SIGMAR1 has been detected in progenitors and mature OL in the rat brain, as well as in SC in the rat sciatic nerve^119,120^. In primary rat OL, the level of SIGMAR1 expression affects OL differentiation by regulating the compartmentalization and transport of lipids^118^. However, the role of SIGMAR1 in OL has not been explored in the context of ALS. Downregulation of either SIGMAR1 or PACS-2 leads to degeneration of neurons and astrocytes, which indicates that MAM integrity is essential for the survival of both cell types^121^. Although these results are intriguing, additional studies are required to determine whether MAM dysfunction in glial cells may directly contribute to the pathogenesis of ALS and HMSN.

## Therapeutic strategies targeting MAMs

Factors modulating MAMs could provide effective targets for therapeutic intervention, by synergistically impacting on the function of both ER and mitochondria. Notably, an agonist of SIGMAR1 (Pre-084) was shown to improve muscle activity and motor performance in pre-symptomatic ALS SOD1 mice^122^. This compound protects motoneurons and extends survival of the treated mice. The same treatment also preserves motoneuron survival and motor performance in the wobbler mice, a model of spontaneous motoneuron degeneration^123^. This finding suggests that pharmacological modulation of MAMs could be beneficial to motoneurons also in models that are not related to mutated SOD1 pathology, and opens therapeutic perspectives for ALS/HMSN diseases involving MAM dysfunction. Recently, another SIGMAR1 agonist, SA4503, was found to reduce cytosolic calcium transients and improve cytoplasmic calcium clearance in cultures of ALS SOD1 motoneurons^124^.

Defects in calcium homeostasis possibly related to MAM dysfunction are a prominent feature in ALS and HMSN diseases^36,46,52,68,125^. Therefore, molecules modulating calcium transfer between ER and mitochondria by activating or blocking IP3R or Ryanodine receptors could be considered as therapeutic agents. However, such an approach would need to be tightly targeted, since an increase in cytosolic calcium can be detrimental. Notably, overexpression of the type 2 IP3R in ALS SOD1 mice has been shown to shorten lifespan of these mice^126^. The use of ER stress inhibitors, such as Salubrinal, could also be a strategy to counteract defects at the level of MAMs. In addition to reducing ER stress, Salubrinal can restore calcium homeostasis in conditions of SIGMAR1 dysfunction^18^. Importantly, daily injections of Salubrinal in ALS SOD1 mice improve their motor functions and extend their survival^86^.

## Conclusion/perspectives

Long-projection neurons such as motoneurons and sensory neurons are particularly affected by ALS and HMSN. This is consistent with the notion that these cells are highly sensitive to any stress, including energy deprivation, which may primarily lead to the degeneration of their axons^2,3,86^. The interplay between ER and mitochondria undeniably plays a pivotal role in maintaining cellular functions essential to neuronal cells. Disruptions of MAM function provoke ER and/or mitochondria stress, with perturbations in calcium homeostasis and energy supply. It is therefore tempting to speculate that perturbations at the level of MAMs could be a starting point for axonal degeneration. However, most of the studies that explored the role of MAM-associated factors linked to ALS and HMSN have used cell lines as model system. Further experiments using primary cultures of sensory or motor neurons as well as *in vivo* models of axonopathies are needed to clarify the role of MAMs in the cell types that are directly relevant to disease.
